# Autologous mesenchymal stem cells offer a new paradigm for salivary gland regeneration

**DOI:** 10.1038/s41368-023-00224-5

**Published:** 2023-05-10

**Authors:** Milos Marinkovic, Olivia N. Tran, Hanzhou Wang, Parveez Abdul-Azees, David D. Dean, Xiao-Dong Chen, Chih-Ko Yeh

**Affiliations:** 1 Department of Comprehensive Dentistry, University of Texas Health Science Center at San Antonio, San Antonio, TX USA; 2 Research Service, South Texas Veterans Health Care System, San Antonio, TX USA; 3 Department of Biomedical Engineering, University of Texas at San Antonio, San Antonio, TX USA; 4 Geriatric Research, Education and Clinical Center, South Texas Veterans Health Care System, San Antonio, TX USA

**Keywords:** Mesenchymal stem cells, Stem-cell niche, Translational research, Stem-cell therapies

## Abstract

Salivary gland (SG) dysfunction, due to radiotherapy, disease, or aging, is a clinical manifestation that has the potential to cause severe oral and/or systemic diseases and compromise quality of life. Currently, the standard-of-care for this condition remains palliative. A variety of approaches have been employed to restore saliva production, but they have largely failed due to damage to both secretory cells and the extracellular matrix (niche). Transplantation of allogeneic cells from healthy donors has been suggested as a potential solution, but no definitive population of SG stem cells, capable of regenerating the gland, has been identified. Alternatively, mesenchymal stem cells (MSCs) are abundant, well characterized, and during SG development/homeostasis engage in signaling crosstalk with the SG epithelium. Further, the trans-differentiation potential of these cells and their ability to regenerate SG tissues have been demonstrated. However, recent findings suggest that the “immuno-privileged” status of allogeneic adult MSCs may not reflect their status post-transplantation. In contrast, autologous MSCs can be recovered from healthy tissues and do not present a challenge to the recipient’s immune system. With recent advances in our ability to expand MSCs in vitro on tissue-specific matrices, autologous MSCs may offer a new therapeutic paradigm for restoration of SG function.

## Introduction

The salivary gland (SG) is composed of a complex network of secretory units and ductal systems that produce and transport saliva, a hypotonic solution containing supersaturated calcium phosphates, lubricants, antimicrobial agents, buffers, and digestive enzymes, which plays a major role in maintaining both oral and overall general health.^1^ There are three pairs of major SGs in mammals: the parotid gland (mainly containing serous acini), the submandibular gland (a mixed gland with mucous acini and serous demilunes) and the sublingual gland (mainly containing mucous acini). In addition, there are a number of minor SGs (mostly mucous acini) in the oral mucosa. Recently, a fourth major SG pair, the tubarial SG, was identified on the posterior nasopharynx and nasal cavity.^2^

The secretory units (i.e., the acini) in human SGs consist of serous and mucous cells which are connected in sequence to the intercalated, striated, and excretory ducts. Salivation is a two-stage secretory process where acinar cells produce a primary saliva, which is subsequently modified by the ductal system. In addition to a watery/aqueous fluid, the serous acini secrete many proteins including amylase, while the mucous acini secrete high molecular weight mucins for lubrication. The acinar products are modified by the ductal system, which secretes K^+^ electrolytes and reabsorbs Na^+^, Cl^−^, and HCO_3_^−^, causing saliva to become hypotonic relative to serum. Overall, the complex secretory function of SGs is tightly regulated by the parasympathetic-muscarinic receptor and sympathetic-adrenergic receptor nervous systems and the circulatory system (i.e. blood flow).

This delicately balanced secretory system can be disrupted by physical injury (e.g. irradiation therapy), autoimmune disorders (e.g., Sjögren’s Syndrome [SS]), medications (e.g., anti-cholinergics, anti-hypertensives, & anxiolytics), or aging, and these disruptions can significantly alter saliva production.^3–5^ As there is a wide variation among patients in baseline salivary flow rates and health status, it is exceedingly difficult to establish a causal relationship between xerostomia and the commonly encountered side-effects of a particular drug class (e.g., antihypertensive drugs).^6,7^ However, there is support for the idea that the number of xerostomia-inducing medications taken by an individual may play a more important role in clinical SG hypofunction and xerostomia than a specific medication by itself.^8–10^

One of the most devastating causes of SG hypofunction is damage induced by ionizing radiation (IR) which occurs in over 60% of patients recieving treatment for head and neck cancers.^11–13^ A second cause of radiation-induced SG damage is radioiodine (I^131^) therapy for thyroid cancer,^14,15^ which also results in clinical mouth dryness.^14,16,17^ Autoimmune-induced SG damage (e.g., SS) also causes acute SG hypofunction and xerostomia.^18,19^ Patients with SG damage experience a profound reduction in quality of life, due to xerostomia (subjective mouth dryness) and hyposalivation (objectively determined by low salivary flow rates), which seriously compromises oral, general, and psychosocial health. In particular, hypofunction of the SG exacerbates oral and systemic diseases (e.g., dental caries, periodontal diseases, mucosal infections, and aspiration pneumonia, etc.), alters the oral microbiome, and significantly impairs digestion (e.g., an inefficient level of mastication, swallowing [i.e., dysphagia], and gustation).^20,21^

Despite a growing body of knowledge regarding SG development and homeostasis, the management of SG hypofunction and dysfunction remains a major clinical challenge. Currently, treatment of SG hypofunction is palliative and mainly consists of moisturizers (e.g., artificial saliva or saliva substitutes) and/or systemic sialagogues (e.g., pilocarpine or cevimeline).^3^ Depending on the severity of SG damage, these synthetic agents often fail to adequately offset the deficit in natural saliva production and relieve symptoms.^5^ Table 1 lists the classes of current and prospective treatments for SG dysfunction and summarizes the challenges and limitations of each.

**Table 1:** Current and prospective therapies for salivary gland dysfunction

Treatment modality	Specific approaches or methods	Known limitations
Palliative (Symptomatic relief)	Saliva substitutes Nutritional counseling	Effectiveness is limited & short-term Oral tissues continue to display deleterious changes Patient compliance is highly variable
Pharmacologic	Sialogogues Immunomodulatory drugs	Effects are systemic Multiple doses/day required Interact with other drugs Patient compliance is highly variable
Gene Therapy	Adeno-associated viruses (AAVs) Oligonucleotides Plasmids	Effect depends on the extent of gland damage Effect can be ablated by immune response Response to treatment is often of limited duration
Cell-based therapies	Salivary gland progenitors Induced pluripotent stem cells (iPSCs) Mesenchymal stem cells (MSCs)	Treatment must be optimized (cell type, route of administration, dose, etc) Cells must engraft and survive for ongoing effect Mechanisms of action are diverse

## Pathogenesis of two major types of SG pathologies

As the aging population continues to grow worldwide, SG dysfunction is expected to increase in prevalence and become a major oral and general health challenge.^10^ Here, we review two major mechanisms that lead to SG hypofunction: (1) the auto-immune disease known as Sjögren’s syndrome (SS) and (2) IR for head and neck cancer.

Sjögren’s syndrome (SS) is a chronic autoimmune disorder characterized by inflammation-driven lymphocytic infiltration (hyperactivated T, dendritic and B cells) and subsequent injury to the epithelium of salivary and lacrimal (exocrine) glands.^19^ SS may also affect other tissues, such as the liver, kidneys, and lungs, or occur secondary to other autoimmune disorders.^18^ Currently, the pathogenesis of SS is only partially understood. Autoimmune attack of the SG epithelium is thought to be triggered by a co-incidence of environmental factors (e.g. latent viral infection) and certain genetic predispositions towards autoimmunity in the host.^19^ Additionally, the ratio of females to males (14:1) affected by SS provides some evidence for the potential involvement of sex hormone expression or X chromosome-linked gene dosage as a factor in the pathogenesis of SS.^22^ Currently, the primary factor driving the development/initiation of SS is thought to be immunological and pro-inflammatory activation of the SG epithelium itself, i.e., “autoimmune epithelitis”,^23,24^ where SG epithelia transform into antigen presenting cells, via increased expression of MHC class II and HLA-DR, and lead to T lymphocyte accumulation.^25,26^ Changes in chemokine profiles have been associated with the development of SS by stimulating adaptive and innate immunity.^24^ Moreover, the pro-inflammatory milieu maintained by the SG epithelium promotes the production of matrix metalloproteinases (MMPs), which progressively degrade the epithelial extracellular matrix and destroy the SG basal lamina.^27–33^

While the association between inflammation and autoimmune attack is certainly an important driver of SS pathogenesis, alterations in intraglandular organization and microenvironment may also play a significant role in creating the conditions for a persistent autoimmune activation. Indeed, evidence from both clinical studies and murine SS models has suggested that changes in SG function may precede the onset of inflammation.^24,32^ For example, studies in NOD mice, an animal model that spontaneously develops SS, show significant changes in SG homeostasis even prior to the onset of inflammation, including decreased nitric oxide synthase production and impaired vasoactive intestinal polypeptide signaling.^34^ Other studies in these mice have shown reduced acinar proliferation and increased MMP activity, resulting in changes in the organization and composition of the epithelium and basal lamina.^35^ MMP-mediated breakdown of the SG extracellular matrix (ECM) leads to the depolarization of acinar cells and a reduction in their secretory activity, independent of inflammation.^32^ Other studies have suggested that breakdown of the ECM may serve as damage-associated molecular patterns (DAMPs), which contribute to autoimmunity by binding pattern-recognition receptors, activating Myd88-dependent signaling, and ultimately promoting chronic B cell stimulation in the SG. For example, small proteoglycans (biglycan [Bgn] and decorin [Dcn]) in the SG ECM are mediators of sterile inflammation and implicated in autoimmunity. While there is no difference in the expression of Bgn and Dcn in the SGs of healthy and SS control mice, Kiripolsky et al. demonstrated that SS mice displayed increased levels of anti-Bgn and -Dcn autoantibodies relative to healthy controls.^36^ Thus, emerging evidence suggests that degradation of the SG-ECM and basal lamina may precede (or take place in parallel with) the onset of inflammation, which is widely considered the ultimate cause of SS development. These findings suggest that potential therapies, which aim to restore a healthy SG microenvironment, may be a very promising, if relatively unexplored, therapeutic avenue.

IR-induced SG damage also has clinically devastating effects, although the precise mechanisms involved have not been fully described. Numerous clinical studies have shown that fractionated doses of IR totaling 40 Gy or more overwhelm the capacity of the SG to repair itself.^37^ Although treatment plans for head & neck cancers frequently employ fractionated dosing schedules to limit adjacent tissue damage, IR therapies still cause moderate-to-severe mouth dryness in many patients.^20^ IR-induced SG damage includes contraction of the SGs, acinar cell atrophy, SG duct dilation, and a reduction in the number of secretory granules.^38–42^ Generally, there is a consensus among researchers that the potential for SG repair is a function of the volume of the gland exposed to radiation, the total dosage received (over time), and whether the surviving progenitor cell population is able to replace the destroyed cells and repair damage to the microenvironment.^43–45^ Previous studies have empirically determined that a mean dose of 26 Gy provides a good balance between eliminating tumor recurrence and preserving SG viability.^46–48^ Recent clinical reports have shown that the irradiation of regions containing the largest number of SG progenitors results in the greatest loss of saliva production.^49^ These findings suggest that stem cell replacement may have potential as a therapy for IR-induced xerostomia.

The SG is a highly organized tissue whose homeostatic processes are characterized by relatively slow turnover of specialized, terminally differentiated cell types. Accordingly, the accumulation of DNA damage is not a major mechanism accounting for its susceptibility to IR-induced damage. Instead, SG damage caused by IR exposure is a dynamic cascade of cellular, paracrine, inflammatory, neuronal, and vascular perturbations that occur in two major phases of response to damage: acute (initial) and chronic (long-term).

In rodent models, the SG acute response to IR (15–30 Gy) begins immediately and includes increased cytoplasmic Ca^2+^ uptake,^50,51^ production of reactive oxygen species (ROS),^52,53^ accumulation of extracellular ATP,^54^ and repair of breaks in double stranded DNA.^55,56^ Somewhat later (3–4 days), a reduction in salivary flow rate and amylase secretion can be measured after IR.^54,57,58^ The acute SG response to IR may reflect an interruption in muscarinic receptor-mediated secretion due to serous acinar cell plasma membrane damage.^44^ Moreover, apoptosis of acinar cells in response to IR may be a major contributor to the functional degeneration of the SG.^45,59,60^ This assertion is supported by studies in transgenic rodent models, which suggest that ablation of apoptotic cascades, such as the p53 pathway^61^ and JNK-signaling^62^ ameliorates SG dysfunction following IR. However, other studies have demonstrated a significant reduction in salivary flow rate during the acute phase with no evidence of a decline in the overall number of acinar cells.^63–65^

The chronic phase typically begins within 3–30 days following IR. In the majority of studies, hyposalivation was sustained for more than 30 days post-IR and the mean onset of fibrosis began around 5 months following initial SG injury.^66,67^ The etiopathogenesis of chronic damage is broadly attributed to acinar cell atrophy and apoptosis and increased senescence of the remaining progenitors, coupled with compromised vascularization and parasympathetic innervation resulting from sustained inflammation and breakdown of the microenvironment.^68–73^ During progression from the acute to chronic response, a host of changes in SG cell polarity, cell-cell contacts, and cytoskeletal organization occurs.^62,74–76^ Recent studies have suggested that IR-induced dysregulation of Yes-associated protein (YAP) nuclear translocation contributes to the post-IR breakdown of glandular homeostasis and function.^77,78^ Eventually, sustained inflammation triggers the replacement of functional SG parenchyma and basal lamina with fibrotic tissue, reducing the vascularization and innervation of the acinar and ductal structures and generally eroding the organizational and functional competency necessary for secretory activity.^39,65,66,79^

Fibrosis is one of the hallmarks of chronic SG injury and, like the acute injury response, is a function of the cumulative dosage and location of the IR source relative to specific functional structures of the gland.^80^ This process not only distorts tissue organization, through the deposition of an abnormal quantity and type of matrix proteins (e.g. fibronectin, collagens, proteoglycans, glycosaminoglycans, etc) in the SG microenvironment, it also scrambles important regulatory cues found in the matrix of the healthy epithelium.^81^ Working with a rodent model, Friedrich et al. demonstrated that the dose-dependent loss of acini caused by exposure to IR is related to an increase in the amount and distribution of ECM proteins, as well as significant compositional changes in the basal lamina.^73^ The study reported that a large dose of IR significantly increased the concentration and altered the distribution patterns of laminin, fibronectin, and collagens (i.e. types III & IV), particularly around acini, granular convoluted tubules and structures of the striated and excretory ducts. In a more recent study, Nam et al. investigated structural and compositional changes occurring in human submandibular glands at 6 months versus 6 years post-IR therapy.^74^ Their findings suggest that IR-induced fibrosis sets in motion matrix-remodeling processes which continue for years in the SG parenchyma following IR-exposure. Relative to healthy controls, disorganization of acinar and ductal structures increased with time post-IR, along with loss of cell-cell tight junctions (evidenced by reduced E-cadherin and zonula occludens-1 (ZO-1) staining). This progressive structural breakdown of the SG from 6 months to 6 years post-IR was accompanied by a significant increase in the density of collagen within the gland.

Recent observations in the pathogenic mechanisms involved in both SS and IR-induced damage point to an important, and relatively under-investigated, role for changes in the SG ECM which drive functional deterioration of the tissue.

## Repairing the damaged SG with regenerative strategies: current approaches and limitations

One major focus of regenerative strategies has been the use of gene therapies to restore secretory function in the damaged gland by augmenting saliva production via increased activity of existing, or synthesis of new, water channels in the damaged SG epithelium. These transmembrane channels increase aqueous permeability and promote glandular secretion in response to osmotic gradients. In both preclinical animal models and Phase 1 clinical trials, viral vectors delivering the human aquaporin-1 gene (*AQP1)* have demonstrated both increased expression of aquaporin-1 protein by SG cells and improved saliva production.^82^ However, while increased salivary flow may alleviate some of the symptoms of xerostomia, it does not entirely restore normal SG function. Moreover, existing gene therapies do not restore the secretory unit per se or repair IR-induced damage to the SG microenvironment. To date, clinical trials assessing the efficacy of *AQP1* gene therapy in IR-induced xerostomia have employed subjects previously treated with radiation doses >15 Gy.^83,84^ Considering the damage caused by ionizing radiation exceeding 15 Gy to SG secretory cells, it is remarkable that *AQP1* gene therapy has shown some degree of effectiveness in these patient cohorts. A related concern is that the vectors carrying the therapeutic transgenes are susceptible to the host immune response. For example, adenoviruses, such as Ad5, have been shown to efficiently transduce salivary epithelium, resulting in a transient, high level of aquaporin expression. However, shortly thereafter, the virus is rapidly cleared by an acute immune reaction.^85–87^ Moreover, the use of viral vectors come with safety concerns involving insertional mutagenesis as well as interference from the host immune response, which has the potential to limit therapeutic dosing and exclude certain patient populations. To overcome these concerns, ongoing clinical trials of *AQP1* gene therapy have employed adeno-associated viruses (AAVs), which are less immunogenic and capable of longer-lasting gene expression relative to adenoviruses.^83,84^

Other genes, such as nerve growth factor and the human neurotrophic factor neurturin (CERE-120), also delivered by AAVs, have alleviated IR induced SG dysfunction in preclinical animal models.^87^ While AAVs have the potential to produce long-term expression of therapeutic proteins in SG cells, a myriad of factors, such as AAV serotype, tissue tropism, transduction efficiency, transgene expression, and pharmacokinetic/pharmacodynamic properties of the protein product, all impact the efficacy and therapeutic duration of individual AAV treatments.^88^ As a result, therapeutic genes delivered by AAVs may provide a transient reduction in symptoms, but not a cure for SG hypofunction.^89,90^

In addition, tissue damaged by advanced SS or IR therapy may be more resistant to gene therapy since the root cause of SG dysfunction involves a major disruption or destruction of the gland, at both the cellular and extracellular matrix (ECM) levels.^5,73^ Because cellular activities in both healthy and damaged tissues are intimately regulated by the composition and architecture of the basement membrane, restoring the major secretory components of the gland necessitates the incorporation of cell and tissue engineering-based approaches. One promising method involves using progenitor (or stem) cells, cultured ex vivo, to produce SG structures (e.g., salispheres; organoids) which can then be transplanted or infused into damaged tissue to initiate the development of a new functional gland in situ.^91^ However, despite numerous reports exploring the feasibility of various cell populations for restoring SG function, an abundant source of SG progenitor/stem cells remains to be identified.^92–95^

In the following sections, we consider the advantages, disadvantages, and limitations of various endogenous cells previously considered for use in SG regeneration and then offer insights into the regenerative potential of autologous, multipotent adult stem cells as an abundant source of cells for SG regeneration and tissue engineering.

## SG development offers insights into relevant regenerative pathways

Comprehensive studies of SG development, many of which have been conducted in rodent submandibular gland (SMG) models, may offer important insights into potential regenerative strategies.^96^ SG development begins prenatally and continues for several weeks after birth in rodents. The process of SG morphogenesis is centered around the patterning and phenotypic specialization of epithelial cells, which is tightly regulated by their microenvironment (niche). By studying these processes, it may be possible to identify cell populations with restorative potential or provide a blueprint for reprogramming stem cells for use in SG repair or regeneration.

**Fig. 1:**
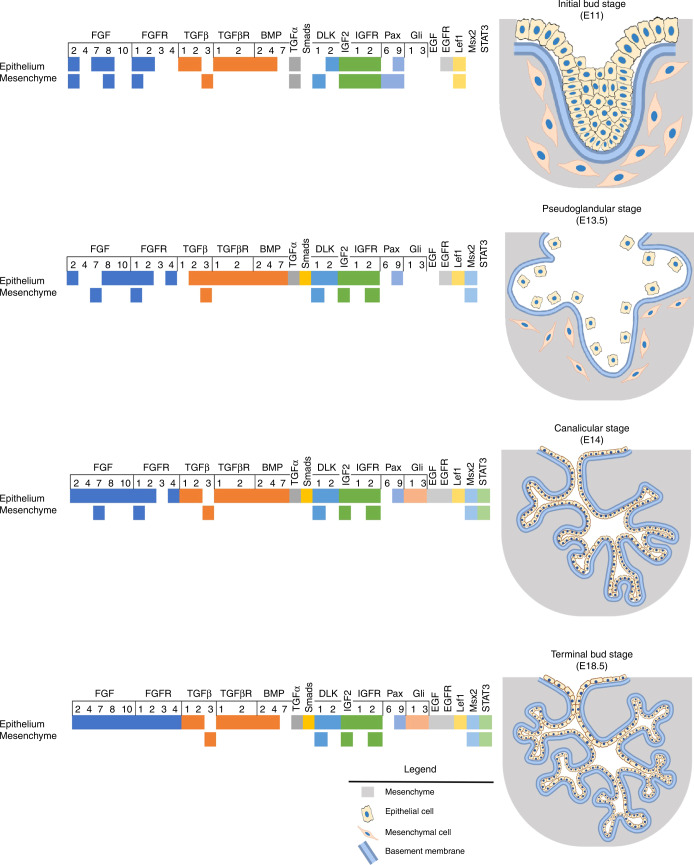
Epithelial-mesenchymal crosstalk during SG development. Studies of SG developmental morphogenesis have demonstrated a complex regulatory relationship between the maturing SG epithelium and the surrounding mesenchyme. The diagram shows signaling pathways that are common to both SG epithelium and mesenchyme and activated or inhibited during various phases of embryonic development. Adapted from previously published gene expression data by Jaskoll & Melnick^227^ and Suzuki et al.^103^ Created using BioRender.com

The prenatal phase of SG development in rodents is generally divided into four main stages: initial bud, pseudoglandular, canalicular, and terminal bud. The initial bud stage begins on embryonic day 11 (E11) as a thickening of the ectoderm-derived oral epithelium forms a bud-like structure within the underlying, neural crest-derived, mesenchyme (Fig. 1). The bud is composed of polymorphic epithelial cells in the center, surrounded by densely packed, columnar peripheral epithelial cells, and held together by E-cadherin protein junctions. Ultrastructurally, both central and peripheral cell types contain round nuclei and mitochondria, numerous polyribosomes, and Golgi, and dilated rough endoplasmic reticuli (rER).^97^ As the bud continues to grow within the mesenchyme, intermingling epithelial and mesenchymal cells begin to assemble an intermediate basement membrane containing ECM proteins and growth factors.^98^

Beginning with the earliest stages, signaling crosstalk between epithelial cells and mesenchymal cells of the developing bud is a critical driver of gland development. For example, the fibroblast growth factor (FGF) signaling pathway consists of four receptors and nearly two dozen ligands. The complete deletion of *FGF10* causes SG agenesis, while heterozygous ablation results in severe hypoplasia.^99–101^ Differential expression of receptors (FGFR1B/2B) and ligands (FGF1/8/13) by the epithelium or receptors (FGFR1C/2C/3/4) and ligands (FGF1/2/3/7/8/10/13) by the mesenchyme is key for patterning of SG branches and growth in all stages of development.^102^ Similarly, the transforming growth factor β (TGFβ) superfamily consists of a number of receptors and ligands, which are also involved in regulating SG growth and morphogenesis from the earliest stages. In the initial bud, expression of TGFβ1/2 ligands is concentrated in the bud epithelium, while TGFβ3 occurs in the mesenchyme. In addition, the corresponding receptors (TGFβR1/2) are chiefly associated with the bud epithelium.^103^ As with FGF signaling, aberrant expression of TGFβ ligands causes significant changes in patterning of the SG branch structure. For example, homozygous ablation of BMP7, a member of the TGFβ superfamily, results in significant defects in branching morphogenesis.^104^ In addition, epithelial-mesenchymal crosstalk involving Notch signaling, which encompasses four receptors (NOTCH1/2/3/4) and four groups of ligands (Jagged [JAG1/2], Delta-like canonical Notch ligand [DLL1/3/4] and Delta-like noncanonical Notch ligand [DLK1/2]), is required for SG development. The suppression of Notch signaling leads to SG dysgenesis, including significant defects in branching morphology.^105^ The expression of Notch ligands in epithelial and mesenchymal cells changes over the course of SG development. However, in the initial bud stage, DLK2 is expressed in the bud, while the surrounding mesenchymal cells express DLK1.^103^

Throughout early gland development, changes in basement membrane composition modulate epithelial cell proliferation and differentiation, which helps direct branching morphogenesis of the developing organ.^106^ For example, ECM heparan sulfates, such as perlecan, direct branching morphogenesis by releasing bound FGF10 to increase mitogen-activated protein kinase (MAPK) activity and promote epithelial cell proliferation in the developing end buds.^107,108^ More broadly, collagens (I, III, IV), laminins, chondroitin sulfates, and fibronectin control branching morphogenesis and specifically collagen IV regulates the differentiation of SG secretory cells.^106,109–112^

By the end of the initial bud stage (~E13), the accumulation of ECM at the bud surface, combined with decreasing expression of E-cadherins at cell-cell junctions, leads to the compartmentalization of groups of cells which become involved in branching, cleft formation, and bud outgrowth.^109–112^ At this stage, most of the epithelial cells, which make up the developing SG, become separated from the supporting mesenchymal cells due to the expression of tight junction proteins (Zonula occludens-1, epithelial cadherins and claudins) and cytokeratin 5.^113–115^ Importantly, this early stage of SG morphogenesis clearly demonstrates that the epithelium and mesenchymal stroma have an intimate relationship during development and suggest a rationale for mesenchymal stem cell (MSC) trans-differentiation as a viable regenerative strategy, as discussed later in this review.^103^

The next step in SG embryological development is the pseudoglandular stage (E13.5) which is characterized by the formation of a lumen containing a central (main) duct and the initial branches of the secondary ducts. These processes are also regulated by the interplay between the rapidly expanding and specializing epithelium and the surrounding mesenchyme.^109,111^ During this stage, the nascent epithelium is composed of peripheral columnar cells, distinguished by the presence of a round nucleus, single Golgi, dilated rER, and absence of secretory granules, surrounding a core of polymorphic cells. These peripheral cells express early pro-acinar markers (i.e., organized E-cadherin junctions),^97^ but don’t produce mucin.^116,117^ Epithelial-mesenchymal cell crosstalk at this stage involves the expression of TGFβ3, which is initially higher in the mesenchyme but later becomes elevated in the epithelium. Changes in Notch signaling begin in the pseudoglandular stage and continue into the canalicular phase of development. These changes include the expression of DLK1 spreading from mainly mesenchymal cells, in the initial bud, to the epithelium distal to the developing bud. Conversely, DLK2 expression, initially highest in the epithelium of the end bud shows a dramatic decrease in this region and increases sharply in the developing ductal cells.^103^

The subsequent canalicular stage completes formation of the lumen, including the secondary and tertiary ducts (E14) and end bud (E15), allowing for transport of fluid from the secretory units to the oral cavity.^98,106,118^ During the latter part of the canalicular stage, epithelial cells in the terminal buds begin to specialize and express acinar markers such as the water channel protein aquaporin-5 (AQP5).^119^ Similarly, the outermost layer of epithelial cells begins differentiation into the myoepithelial lineage and express smooth muscle α-actin.^120^ At this stage, TGFβ3 expression is once again restricted to the mesenchymal cells surrounding the developing SG, whereas expression of TGFβ1/2 ligands and TGFβR1/2 receptors is limited to ducts, luminal cells and terminal regions of the buds.^121^

The terminal bud stage (E18.5) completes the transition of epithelial cells to parenchymal SG cells and includes the apical polarization of cytofilaments along with other morphological changes associated with the production of intracellular secretory granules.^97^ At this stage, TGFβR2 expression is further limited to the duct epithelium,^122^ while DLK1 expression increases in the myoepithelium.^105^ While full maturation of the SG epithelium occurs during postnatal development, the retention of lineage plasticity in both terminally-differentiated SG cells and the surrounding myoepithelium suggests the potential for mesenchymal–epithelial crosstalk as an on-going process in SG homeostasis and repair.

Human SG development follows a similar embryologic model as just described for the rodent and begins with an initial bud stage at gestation weeks 5–6, pseudoglandular stage at weeks 7–8, canalicular stage at weeks 9–10, and terminal bud stage at weeks 19–24.^123–125^

## SG homeostasis and endogenous repair capacity

At maturity, SGs are composed of branching networks containing secretory acini (e.g., serous, mucous or mixed), surrounded by a myoepithelium, that drain their secretions into a ductal system (e.g., intercalated, striated and excretory ducts) that processes, modifies, and transports saliva into the oral cavity.^126^ As described in section 4 above, SG development involves a number of discrete steps where crosstalk between epithelial or ectoderm-derived progenitors and their juxtaposed basement membrane give rise to a complex, branched structure which is highly specialized and contains a number of spatially segregated cell types. Recent studies of SG development and homeostasis have provided important insights into the endogenous regenerative capacity of the tissue, but have stopped short of identifying a multipotent, SG-derived adult stem cell capable of regenerating the entire gland.

**Fig. 2:**
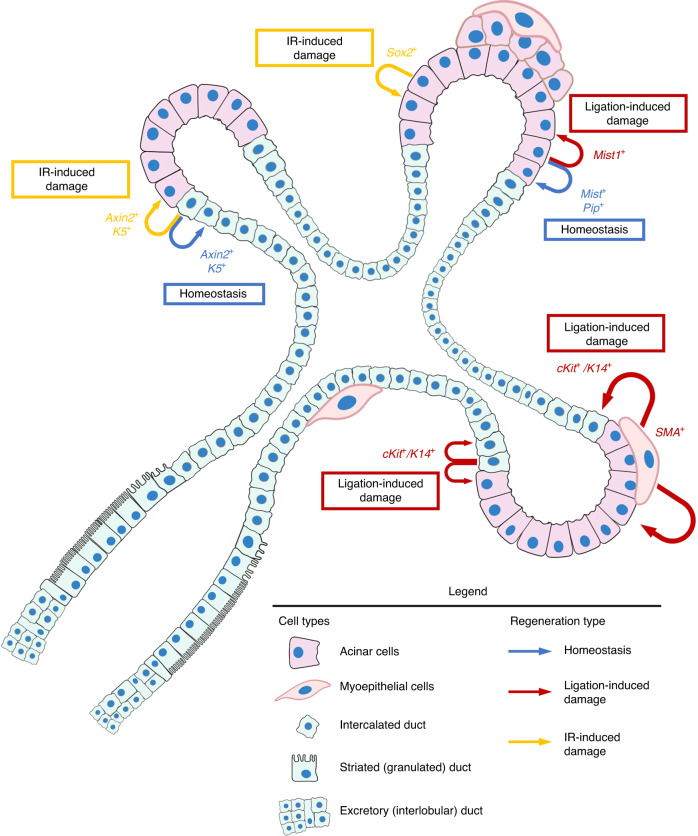
Proposed biomarkers and locations of cells in the adult SG with regenerative capacity during homeostasis or in response to damage. Recent studies have demonstrated that multiple types of SG progenitors have the capacity to maintain homeostasis and regenerate SG function under various conditions. Clonal expansion of terminally differentiated *Mist1*^+^^92^ and *Pip*^+^^127^ acinar cells are involved in homeostatic maintenance and repair of the acinar compartment. The *Mist1*^+^ cells are also activated by ligation-induced damage to form acinar cells.^92^ In the intercalated duct, progenitors expressing *Axin2*^*+*^ or *K5*^*+*^ are responsible for homeostatic maintenance.^128^ However, in response to ligation-induced damage, *cKit*^+^ and *K14*^+^ cells self-renew to repair the intercalated duct or give rise to acinar cells.^129^ SMA-expressing myoepithelial cells also respond to ligation-induced damage by differentiating to form either acinar cells or ductal cells that express *cKit* and *K14*.^129^ Under IR-induced damage conditions, *Axin2*^*+*^ or *K5*^*+*^ ductal progenitors are capable of giving rise to functional secretory acinar cells.^128^ In addition, *Sox2*^+^ acinar cells have been shown to be capable of limited regeneration of the acinar compartment in response to IR-induced damage.^134^ Created using BioRender.com

Models of SG injury have provided many insights into the capacity of resident cells (lineage-restricted progenitors or multipotent stem cells) to repair damage and help define the limits of endogenous regeneration (Fig. 2). Ligation of the excretory duct induces a reversible injury of the acinar parenchyma. When combined with genetic pulse-chase labeling, this approach allows the study of cell populations responsible for regeneration. By use of this method, Aure et al. suggested that a population of *Mist1*-expressing acinar progenitors is responsible for clonal expansion necessary for homeostatic maintenance.^92^ Similarly, Maruyama et al. suggested that a population of acinar cells expressing prolactin-induced protein (Pip) is also capable of clonal expansion for homeostatic maintenance.^127^ In the intercalated duct, Weng et al. reported that both K5^+^ and Axin2^+^ cells behave as lineage-restricted ductal progenitors under homeostatic conditions.^128^ Taken together, these studies show that while SG homeostasis appears to be driven by clonal expansion of terminally differentiated acinar and ductal cells, it is also possible that quiescent stem cells or progenitors are activated in response to injury-induced damage.

To investigate this possibility, Aure et al. used a mouse model to show that a 2-week ligation period resulted in a substantial, but still reversible, loss of acinar cell function.^92^ Genetic labeling suggested that regeneration of the acinar compartment observed in the study was driven by the proliferation (i.e., self-replication) of a surviving population of committed acinar cells rather than activation of multipotent stem cells.^92^ The *Mist1*-expressing acinar progenitors, identified to be involved in homeostatic maintenance, were also shown to be capable of repairing the acinar compartment in response to ligation-induced injury.^92^ To investigate the lineage plasticity of ductal progenitors in SG regeneration, Ninche et al. combined a ligation-induced injury model with in vivo genetic lineage tracing to demonstrate that two non-acinar populations in the submandibular gland, K14^+^/cKit^+^ ductal cells and SMA^+^ myoepithelial cells, *de-differentiate* to a multipotent phenotype before differentiating into acinar cells.^129^ Remarkably, the results showed that >80% of the regenerated acini were derived from these de-differentiated multipotent progenitors. Similarly, Shubin et al. showed that acinar cells under conditions of stress or injury have the ability to display plasticity and transition to a ductal cell phenotype.^130^ Finally, to determine if these SG repair processes have evolved from the developmental pathways reviewed above or represent a distinct regenerative pathway, Minagi et al. employed the duct-ligation model to induce SG damage and then tracked Ki67^+^ cells, along with the expression of AQP5, CK7, tubulin beta 3 (TUBB3) and smooth muscle actin (SMA).^131^ By comparing differences in histology and marker expression, the investigators were able to show that SG development and regeneration represent two distinct processes, driven by marked differences in spatiotemporal patterns of gene expression. Taken together, the evidence from these injury/repair models suggest that some relatively small degree of SG damage can be repaired by an endogenous population of progenitor cells; however, the involvement of a resident population of multipotent stem cells in these regenerative processes cannot be confirmed or ruled out.^132,133^

Unfortunately, the regenerative mechanism which works well for limited SG damage is ineffective for more extensive damage, such as that seen in large numbers of patients who receive IR therapy for head and neck cancer and suffer from SG dysfunction.^68^ Preclinical in vivo rat and mouse models have been developed to directly investigate the ability of endogenous cells to repair IR-induced damage.^20,128^ In these models, IR-induced damage is combined with lineage tracing so that the pathophysiology of human SG dysfunction after IR can be studied.

The results using these models have identified discrete SG regenerative mechanisms that are activated during tissue repair following IR that are independent of the homeostatic and ligation-induced pathways described above. Doses of radiation, which produce clinically-relevant SG damage, induce two temporally distinct pathologies: an initial injury that appears by 30 days post-IR and a second degenerative response by 90 days post-IR. Each elicits its own endogenous repair mechanism. During the initial injury phase, there is a loss of acinar units and ductal cells which are replaced by horizontal division of adjacent acinar and ductal cells.^128,134^ By 90 days post-IR, there is a widespread deterioration of the acinar cell compartment and marked reduction in SG function. In this case, the reparative response begins in acinar progenitors which initiate self-renewal to restore the damaged acini, while ductal progenitors proliferate to maintain duct function and architecture and give rise to new acinar cells.^128,135^ Specifically, K5^+^ and Axin2^+^ ductal cells, shown in Weng et al. to participate in homeostatic maintenance of the intercalated duct, become proliferative in response to IR-induced damage to the acinar compartment and differentiate into functional, secretory acinar cells.^128^ Interestingly, these ductal cells do not participate in the repair of ligation-induced damage to acini. In another related report, Emmerson et al. used an IR-induced injury model to show that a Sox2-expressing population is activated to replenish acinar cells following irradiation.^134^ These results suggest that the identification of an endogenous multipotent stem cell population may only be possible if the SG sustains enough damage to destroy the transiently amplifying population of progenitors or alters the SG niche so much that proliferation of the progenitors is inhibited or no longer possible.

## Identifying SG stem cells

Patients arriving at the clinic with SS or after IR treatment for head and neck cancer, often present with SGs so severely damaged that the capacity of the endogenous regenerative pathways is exceeded. Stem cells have been used in pre-clinical models and clinical trials to treat a wide variety of diseases. As a result, SG-derived stem cells (SG-SCs) have been pursued as very attractive candidates for treating SG dysfunction and the potential to repair damage that cannot be addressed by progenitor-mediated homeostatic mechanisms as described above. Although putative SG-SCs have been isolated and reported to give rise to various cellular components of the gland, reduce inflammation, and ameliorate the acute symptoms of SG dysfunction, they have not been rigorously characterized.^1,136^

Based on international standards for identification and characterization of stem cells, putative SG-SCs must be capable of both long-term self-renewal, or horizontal division without loss of stemness, and differentiation into the mature cell types of a given tissue.^137^ To date, most of the results from SG injury models suggest that SG repair is accomplished through lineage specific progenitors, rather than uncommitted stem cells.

Early efforts to identify the presence of adult stem cells in SG relied on chromatin labeling pulse-chase studies using labeled DNA to distinguish rapidly dividing cells (i.e., likely progenitors), from more slowly dividing, label-retaining cells (LRCs) (e.g., likely multipotent stem cells).^138^ The LRCs identified by this approach were in a state of quiescence outside the replicative cell cycle and in the G0 phase. Use of pulse-chase labeling has been very effective at identifying quiescent stem cells in high turnover tissues such as epidermis, hair follicle, testis, and intestinal epithelium.^138^ However, pulse-chase assays with stem cells in relatively low turnover tissues such as the salivary and prostate glands, lung epithelium, and adipose tissue have provided more ambiguous results.^139^ If SG-SCs indeed serve as a reservoir of regenerative cells, preserved in an off-cycle state to inhibit DNA damage, they would be exceedingly difficult to identify because both quiescent stem cells and terminally-differentiated cells would appear as LRCs. Thus, despite numerous reports of stem cells being identified in both human and rodent SGs, significant questions remain regarding their existence, identity, and properties.^132^ To date, efforts to identify and characterize SG-SCs are impeded by the same issues encountered with low turnover tissues cited above.^133,139,140^

In addition to in vivo lineage tracing of LRCs, SG-SCs have also been identified by the expression of stemness-associated surface markers associated with cells from other tissues or in vitro differentiation assays. Notably, many of these markers were originally identified in studies of development. For example, cKit (Kit; CD117), a receptor tyrosine kinase initially associated with hematopoietic stem cells, has been shown to be expressed by a highly proliferative population of epithelial cells obtained from the SMG that are capable of restoring SG damage in both irradiation- and ligation-induced injury models.^132,133,141^ During SG organogenesis, pathways involving both cKit and fibroblast growth factor receptor 2b (FGFR2b) work together (via respective Akt and mitogen-activated protein kinase [MAPK] pathways) to amplify FGFR2b-dependent transcription, which expands the number of epithelial progenitor cells, increases the proliferation of cKit/K14^+^ progenitors in the distal end buds, and promotes branch formation of the gland.^142^ In contrast, recent evidence from long-term in vivo lineage tracing studies of prenatal through postnatal animals indicate that, at birth, the expression of cKit shifts from cells involved in branching morphogenesis and acinar cell formation, to distributing within a heterogeneous population of at least 2 types of highly-differentiated ductal cells (i.e., intercalated duct and a large duct independent of K14^+^), which remain throughout the life of the animal.^132^ Significantly, there is no evidence supporting a role for cKit^+^ cells in homeostasis or maintenance of any other lineage of SG cells. While different types of cKit^+^ cells appear to be involved in repairing SG damage, it has not been demonstrated that cKit is a reliable marker for SG-SCs. On the other hand, during development, cells expressing cytoskeletal keratins 5 (K5) and 14 (K14) are the progenitors of both acinar and duct cells, which form the ductal compartment in the adult SG and maintain these ductal systems throughout life.^113,132,142,143^

Ascl3, also known as Sgn1, is a member of the mammalian achaete scute (*Mash*) gene family of transcription factors which have been implicated in cell fate specification and differentiation. In mouse major SGs, Ascl3 is initially expressed by a small number of ductal progenitors during embryonic development that give rise to both acinar and ductal cells in mature glands.^144^ More recently, it has been demonstrated that Ascl3^+^ cells are also present in adult glands and retain their progenitor cell properties in vitro.^145^ Ascl3^+^ cells are not capable of differentiating into all types of SG cells, indicating that they are not uncommitted stem cells, but represent an intermediate progenitor cell population. In parallel studies using knockout and cell-specific ablation models in mice, Arany et al. demonstrated that *Ascl3* knockout mice had smaller SGs but secreted saliva normally, contained K5^+^ cells, and were able to repair SG injury due to ductal ligation.^146^ Moreover, lineage tracing studies using Ascl3^+^ spheroid cultures have failed to demonstrate any evidence of co-localization with K5^+^ cells, suggesting that these cells represent two independent populations of adult salivary gland progenitors, rather than multipotent stem cells. In addition, while subpopulations of K14^+^ cells readily proliferate and have lineage-restricted differentiation potential, they have not been demonstrated to serve in a multipotent, regenerative capacity within the adult SG.^132,133^

To date, no reliable cell surface marker has been found which exclusively defines or identifies adult stem cells or, more specifically, SG-SCs. In fact, based on lineage tracing assays and damage repair models, purported markers of SG-SCs have been consistently shown to identify adult progenitor cells or de-differentiated mature cell types with restricted lineage plasticity.^1,37^ These pioneering studies have provided much information describing how SG progenitor cells maintain adult SG tissue homeostasis and their potential for repair/regeneration of damaged SGs. Additional studies are required, however, to identify a reliable biomarker (or biomarkers) for SG-SCs or progenitor cell populations suitable for therapeutic use.

## Ex vivo methods for modeling SG regeneration

SG-specific cell culture methods are required to study the wide variety of cells that make up the SG organ, evaluate regenerative strategies for restoring SG function, and optimize specific cellular activities (i.e., self-renewal, differentiation capacity, & production of trophic and immunomodulatory factors) involved in SG tissue repair.^147^ Various soluble factors have been added to media to mimic SG-specific developmental cues and promote differentiation in vitro. For example, EGF is commonly added to primary cultures of SG cells to promote formation of the ductal lineage.^148^ Similarly, FGFs are used to promote branching morphogenesis; FGF10 is added to stimulate duct elongation and FGF7 to promote terminal bud formation.^149^ Sui et al. have shown that soluble FGF10 promotes the expression of SG markers (MIST1, AQP5, α-SMA, and amylase) by human SMG progenitor cells, while decreasing the expression of K5.^150^ Conversely, inhibitors of Rho kinase (ROCK), epidermal growth factor receptor (EGFR), and TGFβRs have been used to maintain phenotypic expression. For example, ROCK inhibitors have been used to enrich the cKit^+^ and K5^+^ expressing cell populations in SG cell cultures^151^ and promote the expression of c-Met and amylase.^152^ Similarly, EGFR inhibitors have been used in SG cell cultures to retain the acinar cell phenotype and AQP5 expression,^153^ and inhibit K5^+^ and K19^+^ expressing cells of the ductal phenotype.^148^ Finally, different TGFβR inhibitors have been shown to alternately promote ductal^154^ or acinar phenotypes^155^ during in vitro stem cell differentiation.

Aside from differentiation protocols and methods for maintaining various SG cell phenotypes in culture, functional assays are also needed so that the proliferation and differentiation potential of precursor cells (i.e., stem cells and progenitor cells) to the three SG lineages (i.e., acinar, ductal, and myoepithelial) can be measured. However, these assays are typically performed in tissue culture flasks/wells, which don’t contain critical physiologic and physical cues found in the native microenvironment and necessary for observing authentic cell function and behavior.^156–160^

An important advance in this regard was the demonstration that dissociated SG cells could be cultured to form primary salispheres, followed by dissociation, culture, and sorting of single cells by FACS to identify cells expressing surface markers of interest. Subsequently, these cells can be cultured to form secondary spheres and sub-cultured through multiple passages to increase the specific population of cells available for study. As an example, when mouse SG cells expressing CD24^+^ and CD29^+^ were maintained on a Matrigel/collagen matrix, they proliferated (self-renewal) and produced organoids containing cells that when dispersed and sub-cultured in differentiation media produced ductal and lobular organoids. The ductal organoids contained a lumen surrounded by cells that stained positively for CK7 and CK18, while the lobular organoids contained compact, round, lobule-like structures that stained positively for AQP5.^93^ In subsequent studies, the mouse SG cells were divided into three sub-populations, EpCAM^high^ (epithelial cell adhesion molecule), EpCAM^med^, and EpCAM^neg^, by FACS sorting (as above) and cultured on Matrigel:collagen in differentiation media containing EGF, FGF2, insulin, Y-27632, Wnt3a, and Rspo1.^93^ After 24–48 h in culture, the EpCAM^high^ population formed spheres, while the others did not. By 9 days, the spheres formed organoid-like structures that the authors termed “miniglands” which developed lobular structures containing differentiated CK18 positive ductal cells, AQP5 positive acinar cells, and SMAα positive myoepithelial cells. Ultra-structurally, the mini-glands contained serous and mucous acinar cells, based on the presence of electron-dense and less electron-dense secretory vesicles, respectively. Moreover, when the mini-glands were transplanted into SGs, previously damaged by irradiation, saliva production was largely restored. The authors concluded that when individual EpCAM^high^ cells are cultured in Wnt-inducing media, they are capable of producing SG-like structures that contain cells representing all of the lineages found in SGs.^161^ While these results are very encouraging, it is difficult to translate this approach to the clinic because Matrigel is an artificial basement membrane, extracted from murine Englebreth-Holm-Swarm (EHS) tumors, and contains a heterogeneous mixture of ECM components (laminins, collagen IV, entactin, perlecan) and growth factors (TGF-β, EGF, IGF, FGF and others). Importantly, it is highly unlikely that a sufficient number of SG precursor cells can be obtained from damaged SG tissue to reconstruct the native SG microenvironment and support SG cell function and behavior.

Although the use of Matrigel in cell culture has facilitated numerous insights into SG cell behavior and standardized (and reduced) variability between research groups, translation of the results to the clinic and replicating the properties of the tissue-specific (i.e., SG) ECM, which direct SG cell behavior, still need to be addressed. An interesting culture system based on egg white-alginate (EWA) has been developed which provides a simple SG-ECM mimetic with tunable composition and mechanical properties.^162^ By use of another approach, we developed silk fibroin scaffolds as a substitute basement membrane for in vitro maintenance of primary SG epithelial cells and showed that the scaffold supported the formation of aggregates resembling secretory acini.^163^ In addition, we showed that the silk fibroin scaffolds promoted SG cell differentiation and the elaboration of an ECM in vitro which mimicked the native SG niche.

In contrast to these cell culture models which replicate the basement membrane, Su et al. established an organotypic 3D culture model using thin slices of intact human SG which preserves tissue morphology and structure of the native ECM.^164^ The culture system supports the co-culture of acinar, ductal, and myoepithelial cells and maintains cell viability and 3D tissue morphology for up to 14 days. Organ-culture and tissue slice models such as these have significant potential for use in studies examining SG physiology and regeneration ex vivo, but the development and translation of tissue engineering strategies also requires in vitro functional assays that are predictive of regenerative potential.

Salisphere cultures, consisting of spherical, non-adherent clusters derived from single-cell clones of putative SG-derived stem cells, have been used to analyze the regenerative potential of various types of SG-derived cells.^1^ Damage repair models based on transplanted salispheres have shown significant potential in mitigating IR-induced damage,^165,166^ while KIT^+^, CD24^+^/CD29^+^ and CD24^+^/KIT^+^/SCA1^+^ cells derived from salispheres have demonstrated the ability to protect the SG from IR damage in rodent models.^45,70,167^ However, as with any ex vivo method, observations stemming from these cultures need to be interpreted with caution as there are a number of limitations and caveats associated with this method, especially as relates to the purity of the cells, their phenotype, and ambiguity resulting from random cell aggregation.^5,133^ Most notably, the ability to form salispheres, once regarded as a surrogate for self-renewal or regenerative capacity, is now regarded as a general property of SG-derived cell types and not associated with multi-lineage potential.^93,168^

In summary, many issues involving the identification, isolation, validation, and scale-up of adult SG-derived stem cells for the repair or regeneration of damaged SGs must be considered.

## Looking beyond SG stem cells

Multipotent stem cells, derived from non-oral tissues, are an attractive but untapped source of cells for SG regeneration. Over the years, mesenchymal stem cells (MSCs) have been identified in many adult tissues^169^ and display a consistent pattern of cell surface biomarkers including the expression of CD73, CD90 and CD105, but lacking expression of CD11b, CD14, CD19, CD34, CD45, CD79α and HLA-DR.^170^ The most reliable sources of MSCs are adult bone marrow (BM) and adipose (AD) tissues, which have been well characterized for phenotype, quality, self-renewal capacity, and tri-lineage differentiation potential (i.e., osteoblast, adipocyte, and chondrocyte).^171^ Moreover, recent reports have demonstrated that these cells also have the ability to trans-differentiate to a variety of other cell types, including SG epithelial cells.^172–174^

For example, BM-MSCs co-cultured with SG cells have been shown to trans-differentiate to SG acinar cells and express SG markers (e.g., amylase and AQP5) and the tight junction protein claudin-2, which is associated with the mesenchymal-to-epithelial transition.^175,176^ Further, mouse AD-MSCs have been reported to express SG markers when co-cultured with acinar cells separated by a semi-permeable membrane during culture, suggesting the involvement of a diffusible factor in inducing trans-differentiation.^177^ Trans-differentiated BM-MSCs have been used as a source of regenerative cells to repair IR-damaged SG tissue. In Lin et al., BM-MSCs were first labeled with superparamagnetic iron oxide particles and then co-cultured with acinar cells to induce trans-differentiation.^178^ Subsequently, the cells were collected by magnetic sorting and then transplanted into IR treated mice. By day 22 post-transplantation, the SG exhibited functional regeneration, based on salivary amylase activity and saliva production.

Increasing evidence for the trans-differentiation of MSCs comes from studies where MSCs have been engrafted into damaged SG tissue (Table 2). In addition to signs of restored function following IR-damage, such as increased salivary flow rate and glandular mass, these studies also provided histological and genetic evidence for expression of the SG phenotype in transplanted MSCs.^172,179^ In a xenograft model, Lim et al. showed that human AD-MSCs, administered via tail vein injection immediately after IR, homed and engrafted into damaged SG tissues, resulting in improved salivary flow rates, reduced salivation lag times, and higher mucin and amylase production by 12 weeks post-IR.^176^ In addition, by 4 weeks post-IR, TUNEL assays revealed fewer damaged and apoptotic cells in the acinar compartment in animals receiving AD-MSC treatment. Moreover, fluorescence in situ hybridization (FISH) identified the engraftment of human AD-MSCs in SG tissues and the presence of a substantial population of AD-MSCs which co-localized with cells that stained positively for α-amylase, suggesting that at least a portion of the transplanted AD-MSCs had trans-differentiated to the acinar cell lineage in vivo.

**Table 2:** Regenerative potential of MSCs in various models of salivary gland damage

Type of damage	IR Dose (Gy)	MSC source	Donor species	Recipient species	Route of administration	References
SS		BM	Mouse	Mouse	IV	^228^
SS		BM	Mouse	Mouse	IV	^229^
SS		UC	Human	Human	IV	^229^
SS		UC	Human	Human	IV	^230^
SS		UC	Mouse	Mouse	IV	^231^
LG		BM	Rat	Rat	IG	^183^
RI		AD	Rat	Rat	IP	^185^
RI		AD	Human	Mouse	IG	^184^
IR	18	BM	Mouse	Mouse	IV	^179^
IR	15	BM	Mouse	Mouse	IG	^178^
IR	10	AD	Mouse	Mouse	IG	^232^
IR	15	BM	Mouse	Mouse	IG	^181^
IR	15	AD	Human	Mouse	IV	^176^
IR	54 (mean)	AD	Human	Human	IG	^216^
IR	18	AD	Human	Rat	IG	^180^
IR	15	AD	Human	Mouse	IG	^233^
IR	15	AD	Human	Mouse	IG	^234^
IR	13	BM	Rat	Rat	IG	^235^
IR	15	BM	Human	Mouse	IG	^217^
IR	15	BM	Mouse	Mouse	IV	^182^
IR	67 (mean)	AD	Human	Human	IG	^236^

Damage source: *SS* Sjogren’s Syndrome, *LG* Ductal ligation, *RI* radioiodine radiation, *IR* irradiation (gamma radiation), MSC source: *BM* bone marrow, *AD* adipose tissue, *UC* umbilical cord; Route of administration: *IV* intravenous (tail vein), *IG* intraglandular (direct injection into the salivary gland), *IP* intraperitoneal

In a similar xenograft model, another group of investigators reported an increase in salivary flow rate and reduced apoptosis and fibrosis, as well as restored angiogenesis, after transplantation of AD-MSCs directly into the SG immediately after IR (18 Gy) compared to untreated controls.^180^ In another mouse model, autologous BM-MSCs were directly transplanted into the SG at 24 h after IR. By 12 weeks post-transplantation, there was a significant increase in salivary flow rate, as well as histological evidence that administration of BM-MSCs preserved gland morphology and function, reduced apoptosis, and increased microvasculature density compared to untreated IR controls.^181^ Moreover, transplanted BM-MSCs that had been labeled with PKH-26 were detected at 4 weeks post-transplantation and co-localization studies using anti-amylase and confocal microscopy indicated that some PKH-26-positive BM-MSCs had trans-differentiated into SG epithelial cells.

Finally, several studies have demonstrated the potential of intravenous or intraglandular injection of BM- or AD-derived MSC allotransplants for restoring the function of damaged SGs.^179,181–184^ Ahamad et al. elucidated specific mechanisms, related to Ca^2+^ channel influx, which regulate MSC proliferation and differentiation and play a role in increasing AQP5 expression in IR-damaged SG.^182^ Saylam et al. employed intraperitoneal injections of allogenic AD-MSCs in a rodent model of radioiodine-induced SG damage, indicating that animals treated with MSCs showed improved healing and reduced fibrosis at 6 months following radioiodine therapy.^185^ AD-MSCs appeared to protect against the most severe loss of secretory function and facilitated a relatively high degree of structural recovery in damaged SG tissue, as determined by histological analysis at 6 months. Most recently, Kim et al. performed a xenogeneic transplantation study of human AD-MSCs in a murine radioiodine-induced SG damage model.^184^ After investigating a number of potential outcome measures, the study concluded that AD-MSC transplantation following SG injury significantly stimulated tissue remodeling by increasing the density of mucin-laden acinar cells (H&E staining), increasing the expression of AQP5 (an epithelial marker) and CD31 (an endothelial marker), reducing apoptosis (based on TUNEL assay), enhancing salivary flow rate, and restoring the expression of epidermal growth factor and amylase in saliva. By quantifying the expression of human *hALU* in murine SG tissue samples at 16 weeks post-transplantation, the study also firmly established that the *human* MSCs were successfully engrafted.

In spite of substantial progress, major gaps between preclinical studies and an effective and consistent treatment for restoring SG damage in the clinic, using allogenic MSCs, remains to be filled.^186–188^ To date, a relatively small number of stem cell-based clinical trials for xerostomia have been reported. A summary of the trials listed on the *ClinicalTrials.gov* website (https://beta.clinicaltrials.gov/) are summarized in Table 3.

**Table 3:** Clinical trials evaluating the efficacy of MSCs for treating salivary gland damage

Type of damage	MSC source	Transplant Type	NCT #	Trial phase	Country	Year	References
SS	BM	Allogeneic	NCT00953485	½	China	2009	^229^
SS	AD	Allogeneic	NCT04615455	2	Denmark	2020	
IR	AD	Autologous	NCT02513238	2	Denmark	2015	^216,237,238^
IR	BM	Autologous	NCT03743155	2	Spain	2018	
IR	AD	Allogeneic	NCT03874572	1	Denmark	2019	^236^
IR	AD	Autologous	NCT03876197	½	Denmark	2019	
IR	BM	Autologous	NCT04007081	n/a	USA	2019	^217^
IR	AD	Allogeneic	NCT04776538	2	Denmark	2021	

Damage source: *SS* Sjogren’s Syndrome, *IR* irradiation (gamma radiation), MSC source: *BM* bone marrow, *AD* adipose tissue; NCT #: ClinicalTrials.gov identifier

## Challenges associated with allogenic MSC therapy

Reduced SG secretion and a gradual loss of secretory units, due to adipose infiltration into the acinar compartment, are frequently observed with aging due to a loss of stem cell function.^189–193^ Takamatsu et al. showed that the quantity of CD133 + SG epithelial progenitors gradually decline with aging and lose both their differentiation potential and ability to form salispheres in vitro.^194^ In addition, others have reported that the proportion of SG cells exhibiting apoptotic DNA fragmentation increase with aging, along with an increase in p16 activation which suggests changes in the rate of senescence-related cell turnover.^195–197^ Moreover, conditions that damage SG function, such as SS, have been shown to prematurely age putative SG-SCs (or progenitors), which leads to a loss of self-renewal and differentiation capacity, and promote replicative senescence, a reduction in telomerase activity, and an increase in p16 expression.^198^ Together, these changes strongly suggest that even if autologous SG-SCs were available in therapeutically significant numbers, their regenerative capacity is likely to have been severely eroded by aging, injury, or disease. Thus, if SG tissue is damaged beyond repair by endogenous processes, regenerative cell therapies or tissue engineering interventions would require alternative sources of stem cells (e.g., bone marrow or adipose-derived MSCs).

One of the chief benefits of allogeneic MSCs is their potential as an “off the shelf” therapy due their well characterized (i.e., standardized) phenotype, ability to be readily harvested from young and healthy donors, ability to survive cryopreservation, and relatively low immunogenicity. Although somewhat speculative, the low immunogenicity of MSCs has been a key factor in considering their use in the treatment of aging-related diseases, since cells from young donors could be given to patients whose endogenous stem cells have lost much of their self-renewal and/or regenerative capacity.^156,199^ Since MSCs are conceptually viewed as a reservoir of regenerative cells for maintaining tissue homeostasis and healing, they are highly responsive to cues in the local tissue-specific microenvironment (i.e., niche). However, while these signals in the microenvironment regulate self-renewal and/or differentiation activities, they also play a role in MSC phenotypic expression.^200,201^ Recent reports have started to challenge the long-held view that MSCs are immune-evasive and/or immune-privileged, due to their presumed absence of MHC class II or HLA-DR expression. However, these assumptions have been based on experiments where MSCs were cultured on tissue culture plastic (TCP), which is a 2D cell culture environment/surface routinely used for the isolation and study of adherent cells.^202^ Because these culture conditions are artificial and devoid of the complex regulatory signals contained in the tissue-specific (i.e., physiological) microenvironment or niche, MSCs express a phenotype that is not representative of their in vivo behavior. Recently, we found that MSCs cultured on TCP do not express HLA-DR, but when cultured on an ex vivo ECM, which contains many of the attributes of the native tissue-specific MSC niche,^160,199,200,203–206^ HLA-DR expression is strongly upregulated and T cell activity in vitro is stimulated.^207^

Consistent with our observations, allogenic MSCs have recently been shown to promote host immune-rejection and clearance and produce memory T cells specific to the MHC haplotype matching the donor MSCs.^208–210^ As a result, the immune response induced by administration of allogenic MSCs may be the result of a short-term paracrine effect which prevents the donor cells from engrafting and providing any long-term therapeutic benefits (e.g., the replacement and/or repair of damaged cells). These findings may help explain why promising in vitro results in the past have failed to show any new tissue formation by the transplanted allogenic cells and why clinical outcomes using allogenic cells have often failed to meet expectations.^200,211,212^

## Autologous multipotent MSCs as a potential new therapeutic paradigm

To improve the long-term efficacy of stem cell-based therapy, a patient’s own MSCs (i.e., autologous cells) may be preferable. In comparison to MSCs from young donors, those harvested from old (or aged) donors display decreased proliferation rates and differentiation capacities as well as increased numbers of apoptotic and senescent cells, which can limit the efficacy of autologous stem cells in therapeutic applications.^213–215^ Since aged patients are the more likely population to require a regenerative stem cell-based therapy, more information about the changes that occur in MSCs with aging, as well as their tissue-specific microenvironments (niches), is essential.

Recently, the ability of autologous MSCs, harvested from non-oral tissues, to form SG-like structures in vitro and rescue irradiated SG function in vivo has been demonstrated.^216,217^ In 2018, Grønhøj et al., were the first to report the results of a randomized, placebo-controlled Phase 1/2 clinical trial of autologous MSCs for treating IR-induced xerostomia (MESRIX) in 30 patients.^216^ Four months post-treatment, the patients showed a significant improvement in both SG function (unstimulated salivary flow rate and inorganic element secretion/absorption) and patient-reported symptoms of xerostomia, with no detected adverse events. Relative to the placebo group, biopsies of MSC-treated SGs displayed an increase in serous gland tissue, along with corresponding reductions in connective and adipose tissues. Taken together, the results suggested that treatment with autologous MSCs had a regenerative effect on irradiated SG function.

More recently, Blitzer et al. reported the results of a pilot study in which BM-MSCs were successfully harvested from six head and neck cancer patients treated with radiation therapy (i.e., distant from the bone marrow) and having IR-induced SG damage. The MSCs were expanded, characterized, and assessed for their ability to function as an autologous therapy for SG dysfunction.^217^ The results demonstrated that MSCs obtained from patients having received radiotherapy were able to respond to treatment with IFNγ and adopt an immunosuppressive phenotype (i.e., increased expression of IDO, ICAM-1, PD-L1, MHC I, MHC II) and express an anti-inflammatory (& tissue-promoting) secretome (e.g., GDNF, WNT1, R-spondin-1). Importantly, the immunomodulatory and anti-inflammatory characteristics of the MSCs from the cancer patients were similar to age matched controls. Although the study initially aimed to provide pilot data supporting a first-in-human autologous cell therapy trial, the pilot study also evaluated the in vivo regenerative potential of IFNγ-treated human BM-MSCs (i.e., xenografts) transplanted into IR-damaged murine SGs. Compared to untreated controls (i.e., PBS), irradiated murine SGs treated with IFNγ-treated human BM-MSCs showed reduced SG damage, including increased amounts of amylase and mucin expression and reduced collagen fibrosis. Overall, the conclusions drawn from the study suggest that autologous bone marrow-derived MSCs, harvested from patients receiving focused doses of IR, sufficient to treat head and neck cancer and induce SG damage, still retain their regenerative potential for use in therapeutic applications.

However, even if BM-MSCs harvested after radiation therapy retain their immunomodulatory and tissue regenerative properties, the quantity and quality of autologous MSCs from aged donors/patients will display significant variability in their functionality relative to allogeneic MSCs from young healthy donors. Factors such as patient age, tissue source used to harvest the cells (e.g., bone marrow versus adipose tissue), pathophysiological changes resulting from individual patient health history, and pharmacological effects of medications the patients are taking may all impact the therapeutic efficacy and functionality of autologous cells.^90,91,136,138,139^ To overcome these challenges, we developed a three-dimensional (3D) decellularized ECM derived from BM stromal cells (i.e., BM-ECM), as well as other ECMs produced by a variety of tissue-specific stromal cells (e.g., AD-ECM), for use in cell culture. These 3D decellularized matrices provide a more “natural” cell culture environment (versus TCP) and contain many critical biochemical cues, including collagen types I and III, fibronectin, small leucine-rich proteoglycans (e.g., biglycan, decorin), and several major basement membrane components (e.g., perlecan, laminin), that have been shown to play key roles in regulating cell adhesion, migration, proliferation, differentiation, and survival^218–220^ as well as regulate cell behavior in tissues.^204^

Indeed, the results of our studies indicate that mouse and human BM-MSCs, cultured on native decellularized BM-ECM, display enhanced attachment, proliferation, and retention of stem cell properties when compared to culture on TCP.^204–206^ More importantly, when MSCs were obtained from older donors and cultured on “young” ECM, produced by BM-derived stromal cells from young and healthy donors, the expansion of a more “youthful” sub-population of cells from older donors was promoted which exhibited reduced senescence (i.e., intracellular ROS, annexin V and SSEA-4 expression), mitigated expression of senescence-associated secretome markers, and improved regenerative capacity (CFU-F, -OB, and -AD).^199^ In addition, we have reported that ECMs, produced by stromal cells from a variety sources, express tissue-specific differences in protein composition and mechanical and architectural properties, which direct stem cell differentiation to their respective tissue lineage.^158,159^ Recently, we compared the ECMs produced by young versus elderly human BM stromal cells and identified Cyr61/CCN1 as a key regulator of MSC activity which becomes depleted from the BM microenvironment during aging.^201^ These findings point to the important role of tissue-specific cues in the local microenvironment (i.e., niche) that participate in the maintenance of MSC phenotype and direction of stem cell fate. Therefore, it is possible for autologous MSCs, from tissues such as bone or fat and under the appropriate cues in the microenvironment, to trans-differentiate to cells of the SG epithelial cell lineage.

**Fig. 3:**
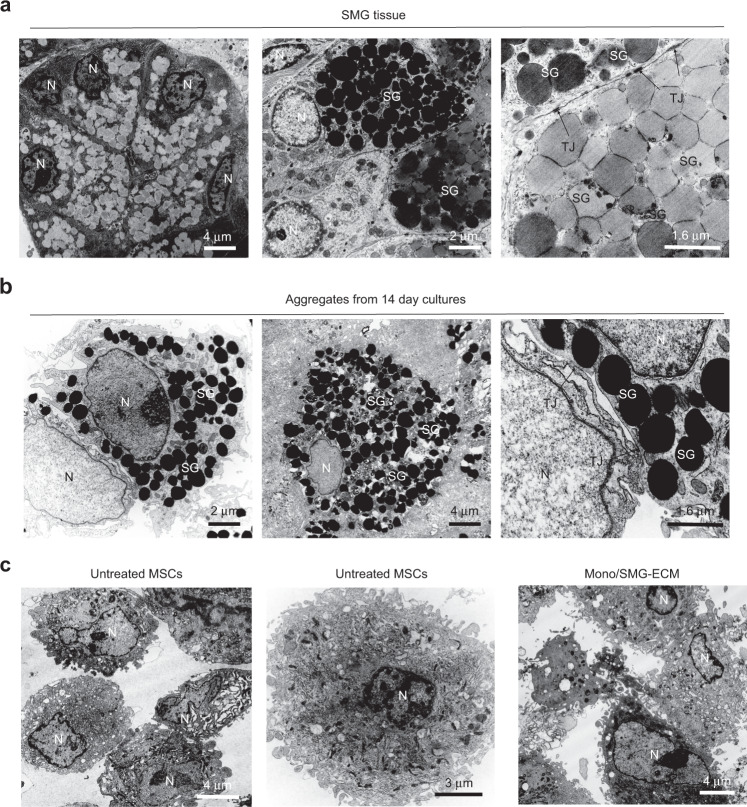
Ultrastructural characteristics of rat SMG tissue and SMG-ECM-treated aggregates were remarkably similar. **a** Transmission electron micrographs of rat SMG (positive control tissue) thin sections. Scale bar is shown in each panel. N: cell nucleus; SG: secretory granule (note the presence of two types of granules, electron dense and less electron dense); TJ: tight junction (identified with thin arrow pointers). **b** Transmission electron micrographs of cell aggregates that formed when BM-MSCs were incubated with SMG-ECM for 1 h and then cultured for 14 days. Note the presence of structures (e.g., electron dense secretory granules; formation of tight junctions; location of the nucleus near the cell membrane) found in rat SMG tissue that can also be seen in the cell aggregates. Scale bar is shown in each panel. **c** Transmission electron micrographs of BM-MSCs that formed a monolayer after treatment with SMG-ECM and culture for 14 days (Mono/SMG-ECM) and untreated BM-MSCs. Scale bar is shown in each panel. Images were selected from original work published by BioMed Central in Tran et al.^221^

By use of this approach, we cultured primary SG epithelial cells on 3D silk fibroin scaffolds and showed that the matrix these cells produce provided an appropriate microenvironment for the differentiation of primary SG cells in vitro.^163^ More recently, we prepared ECM from decellularized rat SMG tissue, and investigated whether this matrix can direct the trans-differentiation of autologous BM-MSCs to the SG epithelial cell lineage in vitro.^221^ We found that culture of BM-MSCs with homogenates of SMG-ECM for 14 days induced the formation of aggregates that expressed SG-specific epithelial cell lineage markers (AQP5, MUC10, KRT14, MIST1) and acinar-associated tight junction proteins (CLDN3 and CLDN10). In addition, the accumulation of glycoprotein/mucin, shown by PAS staining, correlated with the appearance on transmission electron micrographs (TEM) of tight junctions, secretory granule-like structures, and peripheral nuclei, suggesting that the cells were capable of functions associated with differentiated SG cells. Critically, the TEMs in Fig. 3 suggest that only cells that formed aggregates during culture with SMG-ECM expressed markers of the SG epithelial cell phenotype.^221^

**Fig. 4:**
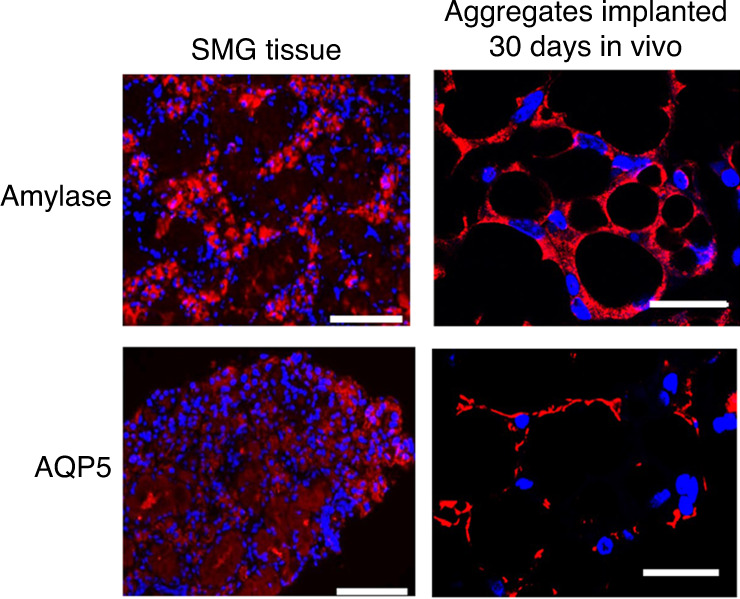
Implantation of aggregates in a sub-renal capsule assay formed SG-like organoid structures in vivo that stained positively for markers of SG differentiation. SMG-ECM-treated aggregates were implanted under the kidney capsule in immunocompromised mice to study organoid development. Kidneys, containing the implants, were harvested after 30 days for histological analysis. After fixation, sectioning, and staining for immunofluorescence microscopy, the presence of amylase and Aqp5, both markers of SG differentiation, were identified in SMG tissue (positive control) and aggregates (formed during 14 days of culture) that had been implanted for 30 days. Staining with nonspecific isotype antibody was used as a negative control (not shown). Scale bar for SMG tissue = 50 µm; scale bar for aggregates implanted for 30 days = 20 µm. Images were selected from original work published by BioMed Central in Tran et al.^221^

To determine if the SG aggregates, formed by the BM-MSCs during culture with homogenates of SMG-ECM for 14 days, were capable of in vivo SG organogenesis, they were implanted under the renal capsule of immunocompromised mice. At 30 days post-implantation, we observed the formation of SG secretory units consisting of ductal and acinar-like cells with tight junction proteins. In addition, these newly formed organoids exhibited strong staining for KRT14, AQP5, CLDN10, and amylase, suggesting that the SG organoids were performing basic functions attributable to SGs (Fig. 4). These results suggested that BM-MSCs were capable of trans-differentiation to the SG epithelial cell lineage when appropriate cues were provided by incubation with decellularized SMG-ECM. Further, the findings also suggest the capability of these aggregates to develop into SG organoids in vivo. We believe that these results open the door to a potential new therapeutic approach based on the use of autologous MSCs from non-oral tissues as an abundant source of SG progenitor cells that could be transplanted for de novo regeneration of damaged SGs.

## Current challenges/future directions

Recent advances in cell lineage tracing and genetic labeling have provided a number of important new insights into the physiology and regenerative potential of the SG. Results employing these methods have identified several potential endogenous repair mechanisms and produced evidence challenging the existence and presumed regenerative function of SG-derived stem cells. In addition, emerging evidence that questions the “immune-privilege” and “immune-evasive” properties of adult stem cells has shifted the balance away from the use of allogeneic MSCs for therapeutic applications. In this context, we suggest that autologous, multipotent stem cells from non-oral tissues, such BM- and AD-MSCs, may have more potential for tissue engineering applications than previously appreciated. Recent studies from our group and others have provided in vitro (Fig. 3) and in vivo (Fig. 4) evidence for the trans-differentiation of MSCs to the SG epithelial cell lineage.

**Fig. 5:**
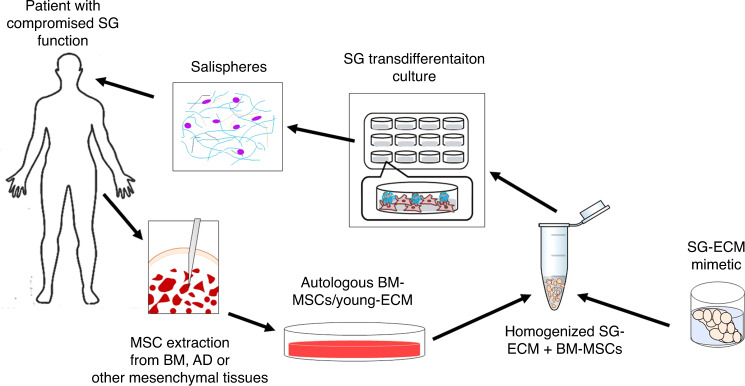
Proposed clinical paradigm for use of autologous BM-MSCs for SG regeneration. MSCs harvested from the bone marrow of patients with SG hypofunction or dysfunction, would be cultured in an environment (i.e., ECM produced by BM cells from young donors [i.e., young BM-ECM]) which promotes the expansion of regenerative subpopulations of autologous MSCs. Once sufficient numbers of MSCs are obtained, they will be combined with a homogenate of decellularized SG-ECM (or, in the future, a mimetic of SG-ECM), which recapitulates components of the healthy SG microenvironment, and induces the trans-differentiation of the MSCs to the SG epithelial cell lineage (i.e., SG progenitors) during culture. After an initial period of culture with the decellularized SG-ECM, followed by the addition of differentiation media and additional time in culture, the cells begin to form SG salispheres, which are transplanted, along with the accompanying ECM, back into the damaged SG of the patient

Based on these observations, we propose a new clinical approach to SG regeneration/repair using autologous MSCs as outlined in Fig. 5. Briefly, autologous MSCs will be obtained from the patient’s own BM or fat tissue and cultured on BM-ECM synthesized by stromal cells from young healthy donors. This will expand and rejuvenate the autologous BM-MSCs which will then be mixed with an homogenate of decellularized SG-specific ECM (or in the future a SG-ECM mimetic), cultured in differentiation media to form SG organoids (salispheres), and subsequently transplanted into the SGs of patients suffering from SG dysfunction or hypofunction.

While the mechanism(s) of MSC trans-differentiation to the SG epithelial cell lineage in vitro and in vivo is not fully understood, MSC-based therapeutic approaches have clearly demonstrated their potential to support/promote tissue repair through a network of complimentary mechanisms. For SS and IR-induced SG damage, MSCs have been shown to modulate inflammation and immunity through multiple pathways (e.g. cell-cell contact, paracrine effects, & secretion of cytokine-containing extracellular vesicles) which suppress long term activation of T lymphocytes, dendritic cells and B cells (Table 2). Moreover, cell-cell contact and MSC-derived paracrine signaling have been shown to moderate the fibroblast inflammatory response by reducing the expression of inflammation-associated adhesion molecules (e.g., ICAM1- and VCAM1) and attenuating the activation of ECM-degrading MMPs.^222–224^ Other cell culture studies have suggested that MSC-derived conditioned media may have the ability to reduce extracellular matrix deposition (i.e. fibrosis) and inflammation in both irradiated human cardiac fibroblasts^225^ and human keloid fibroblasts.^226^ Based on the potential immunomodulatory and anti-fibrotic capabilities of MSCs, it’s possible that administration of MSCs during the early stages of SS or immediately after IR therapy may reduce the loss of SG function and formation of fibrotic tissue and promote repair by the resident cells.

Finally, a number of major challenges remain. A more complete characterization of the various sources of MSCs is needed so that we have a better understanding of their properties and therapeutic potential. More information is also needed regarding the most optimal time to perform MSC transplantation, so that the structure and composition of the remaining SG-ECM contains sufficient microenvironmental cues to direct cell behavior. This “give and take” between the cells and the ECM, before the niche is no longer recognized by the cells, is critical to the repair process, reversing programed cell death, promoting regeneration, and eliminating the formation of fibrotic tissue.

## Conclusions

Our studies have demonstrated that autologous MSCs may be a viable alternative source of stem cells for SG regeneration since they can be expanded, enriched, and rejuvenated in vitro.^199^ We have also shown that BM-MSC differentiation is “plastic” and these cells can be guided to the SG epithelial cell lineage in response to cues in the SG-ECM. However, the translation of autologous MSC-based SG regeneration and repair to the clinic remains in a nascent stage of development. Presently, it appears that three strategies must be combined to make translation of our proposed technology possible. First, a source of autologous stem cells from patients requiring SG regeneration needs to be identified, followed by culture on a “young” ECM to expand and rejuvenate the cell population. Second, the rejuvenated cells need to be exposed to the SG-ECM microenvironment during culture to promote the differentiation and formation of SG organoids or SG epithelial cell progenitors in vitro. Third, procedures for successfully transplanting the SG organoids or progenitor cells into the damaged glands need to be developed so that the timing of transplantation and dosing of organoids can be determined. To achieve these goals, it is essential to integrate cell-to-cell and cell-matrix signaling pathways to gain a comprehensive understanding how MSC trans-differentiation is directed in vitro and to improve the SG-specific microenvironment for in vivo transplantation (Fig. 1).

Historically, the identification of SG-specific biomarkers and the location of these regenerative cell populations in adult SG have been extensively described (Fig. 2). However, it has become clear over time that these putative SG-SCs (and progenitors) are not practical in the clinic since we have been unable to clearly identify SG-SC populations with high regenerative capacity. Interestingly, research emanating from these studies has been useful in selecting subpopulations of multipotent MSCs appropriate for SG regeneration. For example, we have observed that a subpopulation of BM-MSCs enriched in CD133^+^ expression are exquisitely capable of trans-differentiation to the SG epithelial cell lineage in response to incubation with SG-ECM^221^ (Figs. 3 and 4). It is also noteworthy that studies by Takamatsu et al. showed that a subpopulation of CD133^+^ cells in SG decrease with aging.^194^

In addition to the secretory compartment, the SG also maintains an intimate connection with the nervous (e.g., parasympathetic and sympathetic) and vascular systems which support/maintain homeostasis and gland function. During SG regeneration, a major challenge involves connecting the highly differentiated epithelial components with the vascular and nervous systems. To address these gaps in our knowledge, ongoing efforts are pursuing more efficient MSC trans-differentiation strategies that must eventually be combined with advanced tissue engineering approaches to accomplish re-vascularization and re-innervation of the SG.
